# Allopregnanolone in the bed nucleus of the stria terminalis modulates contextual fear in rats

**DOI:** 10.3389/fnbeh.2015.00205

**Published:** 2015-08-04

**Authors:** Naomi Nagaya, Gillian M. Acca, Stephen Maren

**Affiliations:** ^1^Texas A&M UniversityCollege Station, TX, USA; ^2^Institute for Neuroscience, Texas A&M UniversityCollege Station, TX, USA

**Keywords:** fear conditioning, allopregnanolone, sex differences, context, freezing

## Abstract

Trauma- and stress-related disorders are among the most common types of mental illness affecting the U.S. population. For many of these disorders, there is a striking sex difference in lifetime prevalence; for instance, women are twice as likely as men to be affected by posttraumatic stress disorder (PTSD). Gonadal steroids and their metabolites have been implicated in sex differences in fear and anxiety. One example, allopregnanolone (ALLO), is a neuroactive metabolite of progesterone that allosterically enhances GABA_A_ receptor activity and has anxiolytic effects. Like other ovarian hormones, it not only occurs at different levels in males and females but also fluctuates over the female reproductive cycle. One brain structure that may be involved in neuroactive steroid regulation of fear and anxiety is the bed nucleus of the stria terminalis (BNST). To explore this question, we examined the consequences of augmenting or reducing ALLO activity in the BNST on the expression of Pavlovian fear conditioning in rats. In Experiment 1, intra-BNST infusions of ALLO in male rats suppressed freezing behavior (a fear response) to the conditioned context, but did not influence freezing to a discrete tone conditioned stimulus (CS). In Experiment 2, intra-BNST infusion of either finasteride (FIN), an inhibitor of ALLO synthesis, or 17-phenyl-(3α,5α)-androst-16-en-3-ol, an ALLO antagonist, in female rats enhanced contextual freezing; neither treatment affected freezing to the tone CS. These findings support a role for ALLO in modulating contextual fear via the BNST and suggest that sex differences in fear and anxiety could arise from differential steroid regulation of BNST function. The susceptibility of women to disorders such as PTSD may be linked to cyclic declines in neuroactive steroid activity within fear circuitry.

## Introduction

In the U.S., the lifetime prevalence of trauma- and stress-related disorders is 60% greater for women compared to men (Kessler et al., 2005). Indeed, posttraumatic stress disorder (PTSD) is twice as likely to occur in women. Sex differences in fear and anxiety have also been reported in non-human species. One well-established behavioral paradigm that models aspects of PTSD, Pavlovian fear conditioning, has revealed sex differences in rodents (Maren et al., 1994; Markus and Zecevic, 1997; Aguilar et al., 2003; Kudo et al., 2004; Wiltgen et al., 2001; Chang et al., 2009; Gresack et al., 2009; Barker and Galea, 2010; Daviu et al., 2014) as well as in humans (Milad et al., 2006; Grillon, 2008; Lebrón-Milad et al., 2012). In this procedure, a neutral conditioned stimulus (CS, tone) that has been paired with an aversive unconditioned stimulus (US, footshock) comes to elicit conditioned fear responses, including freezing, increases in acoustic startle, and changes in heart rate and blood pressure (LeDoux, 2000; Maren, 2001; Fanselow and Poulos, 2005). After fear conditioning, male and female rats show a dramatic sex difference in levels of contextual freezing: females express significantly lower levels of freezing in the conditioning context compared to males (Maren et al., 1994; Markus and Zecevic, 1997; Barker and Galea, 2010). Interestingly, males and females exhibit similar levels of freezing during conditioning and during the expression of fear to the discrete CS (Maren et al., 1994; Markus and Zecevic, 1997; Barker and Galea, 2010).

One potential neural substrate for this sex difference in contextual fear is the bed nucleus of the stria terminalis (BNST). The BNST receives input from multiple limbic structures involved in emotional processing and sends output directly to the hub of the hypothalamic-pituitary-axis, the paraventricular nucleus of the hypothalamus (Crestani et al., 2013). BNST lesions or inactivation selectively impair freezing to shock-associated contexts, but not auditory CSs (Hammack et al., 2004; Sullivan et al., 2004; Resstel et al., 2008; Zimmerman and Maren, 2011). In addition, BNST inactivation reduces light- (Walker and Davis, 1997) and corticotropin-releasing hormone (CRH)-enhanced (Lee and Davis, 1997) startle, two forms of startle potentiation argued to reflect contextual fear. BNST lesions also reduce fear to long-duration auditory CSs that mimic the temporal properties of contextual stimuli (Waddell et al., 2006). Moreover, recent optogenetic approaches have shown that discrete neural circuits within the BNST are involved in regulating responses to aversive contexts (Jennings et al., 2013; Kim et al., 2013). Collectively, these data suggest that the BNST is central for the expression of conditional fear responses, including freezing, to aversive contexts.

Given its role in reproductive (Claro et al., 1995; Liu et al., 1997) and maternal (Numan and Numan, 1995, 1996) behaviors, the BNST is a prime site for hormonal modulation. BNST neurons have receptors for estrogen (Laflamme et al., 1998), progesterone (Auger and De Vries, 2002), and androgens; levels of estrogen and progesterone receptors are similar between male and female rats whereas levels of androgen receptors are greater in males (Roselli, 1991). Indeed, hormonal modulation of BNST activity in fear conditioning has been implicated by studies of intact cycling females and ovariectomized females treated with gonadal steroids. For example, natural fluctuations in ovarian steroids across the estrous cycle are associated with differences in the expression of contextual fear such that rats in proestrus, when progesterone and estradiol levels are highest, show the lowest level of fear compared to males and females in estrus (Markus and Zecevic, 1997). Moreover, systemic administration of either progesterone or its neuroactive metabolite, allopregnanolone (ALLO), impairs CRH-enhanced increases in acoustic startle, a form of startle that is mediated by the BNST (Toufexis et al., 2004). A potent allosteric potentiator of GABA_A_ receptors (Majewska et al., 1986), ALLO has been linked to reduced anxiety in a variety of other behavioral paradigms in which female rodents appear less anxious than males (Frye et al., 2000; Hughes et al., 2004), including the elevated plus maze (Bitran et al., 1991), the defensive burying test (Picazo and Fernández-Guasti, 1995), the light/dark transition test (Wieland et al., 1991), and the open field test (Wieland et al., 1991). Although ALLO can be synthesized within the gonads, adrenal glands, and brains of both male and female rats, it is found in higher concentrations in female brains and plasma (Corpéchot et al., 1993). In females, ALLO levels in whole brain and plasma are 8- to 10-fold higher than in males (Purdy et al., 1990; Corpéchot et al., 1993; Cheney et al., 1995).

Collectively, this work suggests that ALLO may regulate fear and anxiety by modulating neuronal activity in the BNST. To explore this question, we assessed whether augmenting ALLO activity in the BNST of male rats would decrease contextual freezing (Experiment 1) and, conversely, whether reduction of ALLO activity in the BNST of female rats would increase contextual freezing (Experiment 2). Males received intra-BNST infusions of ALLO whereas females received infusions of either an ALLO synthesis inhibitor, finasteride (FIN; Finn et al., 2006), or a selective antagonist, 17-phenyl-(3α,5α)-androst-16-en-3-ol (17-PA; Mennerick et al., 2004; Kelley et al., 2007). Importantly, this set of experiments examines whether hypothesized sex differences in endogenous intra-BNST ALLO (i.e., low endogenous ALLO activity in males and high endogenous ALLO activity in females) contribute to the contextual fear phenotype in each sex. We now show that manipulations of ALLO activity within the BNST affect the expression of contextual fear in both male and female rats, a finding that supports neuroactive steroid modulation of the BNST in fear and anxiety.

## Materials and Methods

### Animals

Male and female Long-Evans rats (200–224 g; Blue Spruce) were obtained from a commercial supplier (Harlan Laboratories, Indianapolis, IN, USA). At the time of arrival, rats were individually housed in clear plastic cages; males and females were kept in separate rooms. Lights were maintained on a 14:10 h light:dark cycle (lights on at 7:00 A.M.) with access to food and water *ad libitum*. Upon arrival, each rat was handled for 20 s each day for five consecutive days to acclimate to the experimenter. All experiments were carried out in accordance with guidelines approved by the Institutional Animal Care and Use Committees at Texas A&M University.

### Behavioral Apparatus

All behavioral procedures occurred in eight identical observation chambers (30 × 24 × 21 cm; Med Associates, St. Albans, VT, USA) composed of aluminum sidewalls and Plexiglas ceiling, rear wall, and front door. One sidewall contained a speaker for CS delivery and the other contained an incandescent house light. Chamber floors consisted of 19 stainless steel rods (4 mm in diameter) spaced 1.5 cm apart (center to center) for footshock US delivery. The rods were wired to a shock source and solid-state grid scrambler (Med Associates). Beneath the rods was a removable stainless steel tray. In addition, each chamber was positioned in a sound-attenuating cabinet equipped with a ventilation fan to provide background noise (65 dB).

The behavioral procedures were conducted in two distinct contexts. For “context A” (conditioning and context test), the chambers were cleaned with 1% acetic acid; the pans beneath the floors were also rinsed in acetic acid. The house lights (15 W) in the chambers were lit and the cabinet doors were left open. White fluorescent room lights, the computer monitor, and cabinet fans were on. Animals were transported to and from the vivarium in white plastic boxes. For “context B” (tone test), the chambers were cleaned with 1% ammonium hydroxide. The house lights were turned off and the cabinet doors were closed. Red fluorescent lights illuminated the room, the computer monitor was turned off, and the cabinet fans were on. Animals were transported to and from the vivarium in black plastic boxes.

Locomotor activity was measured by recording the displacement of the load cell platform located underneath each chamber (Maren, 1998). Prior to the experiment, each load cell amplifier was calibrated to a fixed chamber displacement with the output of each amplifier set to a specific gain to detect immobility. The output of the load cell amplifier was digitized such that one observation every 200 ms for each rat was recorded via the Threshold Activity software (Med Associates). Freezing behavior was derived from the locomotor activity as previously described (Maren, 1998).

### Surgery

Male rats were anesthetized with ketamine (100 mg/kg body weight; i.p.) and xylazine (10 mg/kg body weight; i.p.) and treated with atropine sulfate (0.04 mg/kg body weight, i.p.). Female rats were anesthetized with ketamine (60 mg/kg body weight; i.p.) and xylazine (8 mg/kg body weight; i.p.) and treated with atropine sulfate (0.04 mg/kg body weight; i.p.). After shaving the top of the head, each rat was secured in a stereotaxic apparatus (David Kopf Instruments, Tujunga, CA, USA). The scalp was incised and retracted. Head position was adjusted to align lambda and bregma in the same horizontal plane. Small burr holes (1 mm in diameter) were drilled bilaterally into the skull for placement of guide cannulae (stainless steel, 26-gauge, 9 mm below pedestal; Plastics One, Roanoke, VA, USA) directed towards the BNST (0.0 mm AP and 2.7 mm ML relative to bregma, −6.9 mm DV from dura at a 10° angle from vertical towards the midline). In addition, three burr holes were drilled for the placement of anchoring screws. After implantation of guide cannulae, dental acrylic was applied to affix the cannulae to the skull. After surgery, dummy cannulae (33-gauge, 9 mm with 1-mm projection; Plastics One) were inserted into the guide cannulae. Rats were allowed to recover for 1 week before behavioral procedures. To habituate the animals to the infusion procedures, rats were individually transported in white 5-gal buckets lined with bedding to the infusion room (within the vivarium) and received dummy changes on two separate days during the recovery period.

### Vaginal Smears

Vaginal smears were obtained from all female rats for at least eight consecutive days to ensure normal cycling; estrous cycle phase was not used as a variable in the analysis due to sample size. To track estrous cycle phase, vaginal smears were taken daily with cotton swabs moistened with distilled water between 8:00 and 10:00 A.M. starting the day of surgery and continuing throughout the behavioral procedures (allowing for at least 1 h between smears and behavior). Cells were visualized under a light microscope at 100× and characterized according to Goldman et al. (2007). One rat with an irregular cycle was excluded from the analysis.

### Drugs

All drugs were prepared in a 30% (w/v) solution of the complexing agent hydroxypropyl-β-cyclodextrin (VEH; Sigma-Aldrich, St. Louis, MO, USA) in purified water. Allopregnanolone or (3α,5α)-3-hydroxy-pregnan-20-one (ALLO; R&D Systems, Minneapolis, MN, USA) was solubilized in VEH (8 mg/ml). The 5α-reductase inhibitor commonly known as finasteride, (5α,17β)-N-(1,1-dimethylethyl)-3-oxo-4-azaandrost-1-ene-17-carboxamide (FIN; Sigma-Aldrich), was solubilized in VEH (10 mg/ml). The steroid antagonist, 17-phenyl-(3α,5α)-androst-16-en-3-ol (17-PA; R&D Systems) was solubilized in VEH (3.5 mg/ml). Infusions were made with 10-μl Hamilton syringes mounted into an infusion pump (KD Scientific, Hollistan, MA, USA) and connected to internal cannulae (33-gauge, 9 mm with 1-mm projection; Plastics One) with either polyethylene tubing (PE-20, Braintree Scientific, Braintree, MA, USA) for VEH or polytetrafluoroethylene tubing (PTFE; 28-gauge, SAI Infusion Technologies, Lake Villa, IL, USA) for drugs. PTFE tubing was used to minimize drug loss due to nonspecific binding.

## Procedures

### Experiment 1: Effects of ALLO on the Expression of Contextual and Cued Fear in Male Rats

For the ALLO study, 31 male Long-Evans rats were housed and cannulated as described above. On Day 1, rats were transported to the laboratory and placed in the conditioning chambers (context A) for training. After a 3-min baseline period, rats were presented with five tone (CS; 10 s, 80 dB, 2 kHz)-shock (US; 2 s, 1 mA) pairings in which the tone was immediately followed by the shock. There was a 1-min inter-trial interval (ITI) between each tone-shock pairing followed by a 1-min wait period after the final shock. On Day 2, 24 h later, squads of 4 rats were transported to the infusion room in white 5-gal buckets lined with bedding. Rats received bilateral intra-BNST infusions (0.25 μl at 0.25 μl/min) of either VEH (*n* = 15) or ALLO (2 μg/side; *n* = 16). The dosage and timing of ALLO infusions were based on previous reports of behavioral effects resulting from intracranial infusions (Bitran et al., 1991; Akwa et al., 1999; Engin and Treit, 2007); BNST infusion volumes were based on previous work from our laboratory (Zimmerman and Maren, 2011). After the 1-min infusion, internal cannulae were left in place for 2 min to allow for drug diffusion and then replaced with clean dummy cannulae. Context testing began ten minutes after the start of infusions. Rats were placed in the conditioning chambers (context A) for a 10-min context test in which no tones or shocks were delivered. On Day 3, rats were infused in the same manner with the same drug as on Day 2 and, at 10 min after the start of infusions, were placed in a novel context (context B) for a tone test. The tone test consisted of a 3-min baseline period followed by four tone (CS; 10 s, 80 dB, 2 kHz) presentations with a 1-min ITI and a 1-min wait period after the last tone. The conditioning and testing procedures (including the order of context and tone tests) were patterned after the experimental designs used in many of our studies (Maren et al., 1997; Maren, 1998, 1999; Zimmerman and Maren, 2011), including work revealing sex differences in the expression of contextual fear (Maren et al., 1994).

### Experiment 2: Effects of FIN and 17-PA on the Expression of Contextual and Cued Fear in Female Rats

Seventy-six female Long-Evans rats were housed and cannulated as described above. On Day 1, rats were transported to the laboratory, placed in the conditioning chambers (context A) and trained in the same manner as in Experiment 1. On Day 2, squads of 8 rats were transported to the infusion room in white 5-gal buckets lined with bedding. Rats received bilateral intra-BNST infusions (0.25 μl at 0.25 μl/min) of VEH, FIN (2.5 μg/side), or 17-PA (0.875 μg/side). The doses of FIN and 17-PA were based on previous reports (Frye and Vongher, 2001; Rhodes and Frye, 2001; Frye and Walf, 2002; Walf et al., 2006; Kelley et al., 2007; Svensson et al., 2013). After the 1-min infusion, internal cannulae were left in place for 2 min to allow for drug diffusion and then replaced with clean dummy cannulae. Animals receiving FIN infusions (and a subset of VEH controls) were returned to their home cages for 2 h prior to retrieval testing to allow sufficient time for 5α-reductase inhibition (Rhodes and Frye, 2001; Frye and Walf, 2002; Walf et al., 2006). Rats in the 17-PA group (and a subset of VEH controls) were tested 10 min after their infusions (Svensson et al., 2013). For the context testing, rats were transported to the conditioning chambers (context A) for a 10-min context test as described in Experiment 1. On Day 3, rats were infused with the same drug as on Day 2 and were transported to a novel context (context B) for a tone test as described in Experiment 1. One rat from the FIN group was excluded due to acyclicity and a squad of rats (4 VEH and 4 17-PA) was excluded due to an equipment malfunction. Data from VEH controls for the FIN and 17-PA groups were collapsed for analysis as they did not differ. This left group sizes of: VEH (*n* = 26), FIN (*n* = 15), and 17-PA (*n* = 12).

### Histology

After behavioral testing, all rats were overdosed with pentobarbital (100 mg/kg) and transcardially perfused with 0.9% saline followed by 10% formalin. Brains were rapidly dissected and post-fixed in 10% formalin for 24 h before transfer to a 30% sucrose-formalin solution. Brains were sectioned at 40 μm on a cryostat at a constant temperature of −20°C and mounted on subbed slides with 70% ethanol. Sections were stained with 0.25% thionin to visualize cannulae placements within the BNST.

### Data Analysis

In all experiments, the percentage of freezing behavior was averaged across 1-min blocks in each behavioral session (the 10-s CS periods were excluded from the analysis of the conditioning and tone test sessions) and analyzed using analysis of variance (ANOVA). We hypothesized that across the context tests, ALLO would reduce freezing in males and FIN and 17-PA would increase freezing in females. Therefore, *a priori* planned comparisons using Fisher’s PLSD test were computed after significant effects in the ANOVA. All data are represented as means ± SEMs.

## Results

### Intra-BNST ALLO Infusion and Contextual Fear in Male Rats

#### Histology

**Figure 1:**
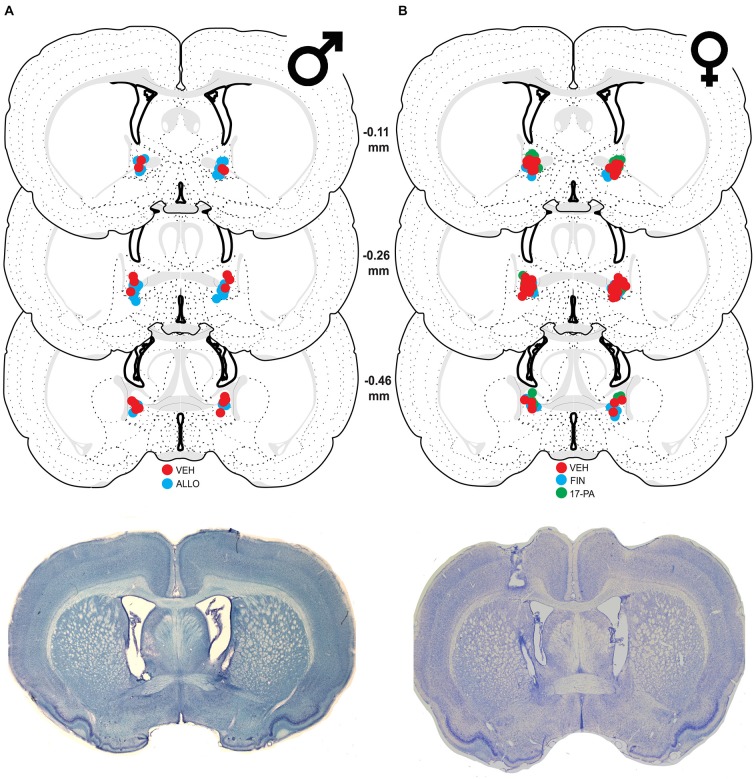
**Schematic coronal sections showing cannula placements in the bed nucleus of the stria terminalis (BNST).** Cannula placements are indicated for infusions of vehicle (VEH), allopregnanolone (ALLO), finasteride (FIN), and 17-phenyl-(3α,5α)-androst-16-en-3-ol (17-PA). Representative thionin-stained sections are shown below each set. **(A)** Infusion sites for VEH (red circles) and ALLO (blue circles) in male rats with photomicrograph of a representative section. **(B)** Infusion sites for VEH (red circles), FIN (blue circles), and 17-PA (green circles) in female rats with photomicrograph of a representative section. Coronal brain section images adapted from Swanson (2003).

Of 31 male rats receiving cannulae, nine were excluded because their cannulae were not centered in the BNST. This yielded the following group sizes: VEH (*n* = 9) and ALLO (*n* = 13). As shown in Figure 1A, the majority of cannula placements were centered at 0.26 mm caudal to bregma, although placements both rostral and caudal to that level were similarly represented.

#### Behavior

**Figure 2:**
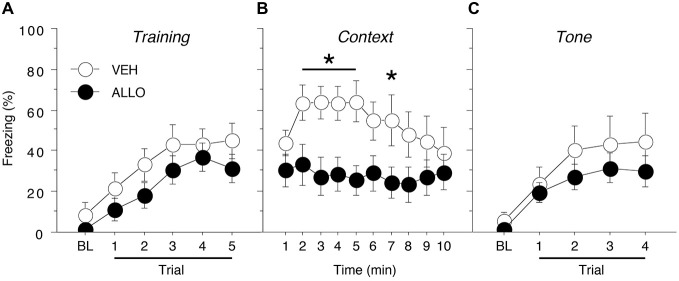
**Conditioned freezing in male rats receiving pre-test infusions of ALLO into the BNST. (A)** Mean percentage of freezing (±SEM) during the five-trial training session (data are shown with a 3-min pre-trial period followed by five tone-shock pairings). Freezing was quantified before the first conditioning trial (baseline, BL) and during the 1-min period after each conditioning trial. **(B)** Mean percentage of freezing (±SEM) to context over 10 min 1 day after training. **(C)** Mean percentage of freezing (± SEM) to four auditory conditioned stimulus (CS) presentations in a novel context 2 days after training. Freezing was quantified before the first tone trial (baseline, BL) and during the 1-min period after each tone trial. *p < 0.05 ALLO vs. VEH.

Acquisition of fear conditioning did not differ between male groups assigned to receive infusions of VEH or ALLO (Figure 2A). Levels of freezing were low (<10%) prior to the start of training and increased across the five training trials similarly for all animals. A repeated measures ANOVA with a between-subjects variable of drug group (VEH or ALLO) showed a significant main effect of trial number (TRIAL) on the level of freezing expressed (*F*_(5,100)_ = 18.0, *p* < 0.0001) but neither the main effect of drug group (*F*_(1,20)_ = 1.88, *p* = 0.19) nor the interaction between drug group and trial number (*F*_(5,100)_ = 0.30, *p* = 0.91) was found to be significant. Thus, all male rats acquired conditioned fear at similar rates and levels prior to their drug manipulations.

Intra-BNST ALLO infusions 10 min prior to testing resulted in a decreased level of freezing to the conditioned context for male rats (Figure 2B). A repeated measures ANOVA with a between-subjects variable of drug group (VEH or ALLO) showed that intra-BNST infusion of ALLO suppressed freezing throughout the 10-min context test. The analysis revealed significant main effects of drug group (*F*_(1,20)_ = 5.22, *p* < 0.05) and time (min 1–10; *F*_(9,180)_ = 2.08, *p* < 0.05). In addition, there was a significant interaction between drug group and time (*F*_(9,180)_ = 1.99, *p* < 0.05). Planned comparisons (*p* < 0.05) of the average freezing during each minute of the context test revealed that ALLO significantly reduced freezing during minutes 2–5 and minute 7 of the test (Figure 2B). These data indicate that acute ALLO administration in the BNST suppressed the expression of contextual fear in male rats.

By contrast, pre-test infusion of ALLO into the BNST did not affect conditional freezing during the tone test (Figure 2C). A repeated measures ANOVA with a between-subjects variable of drug group (VEH or ALLO) and a within-subject variable of trial number (TRIAL) revealed a main effect of trial (*F*_(4,80)_ = 16.9, *p* < 0.0001), but there was neither a significant main effect of drug group (*F*_(1,20)_ = 0.97, *p* = 0.34) nor an interaction between drug group and trial (*F*_(4,80)_ = 0.50, *p* = 0.73). These data indicate that acute ALLO administration in the BNST produces a selective reduction in contextual freezing in male rats.

### Intra-BNST Infusion of FIN or 17-PA and Expression of Contextual Fear in Female Rats

#### Histology

Of the 67 cycling female rats, 14 were excluded because their cannulae either missed the target or were not patent (resulting in a unilateral infusion). This yielded the following group sizes: VEH (*n* = 26), FIN (*n* = 15), and 17-PA (*n* = 12). As shown in Figure 1B, the majority of cannula placements were centered 0.26 mm caudal to bregma although there were many placements rostral and caudal to that level.

#### Behavior

**Figure 3:**
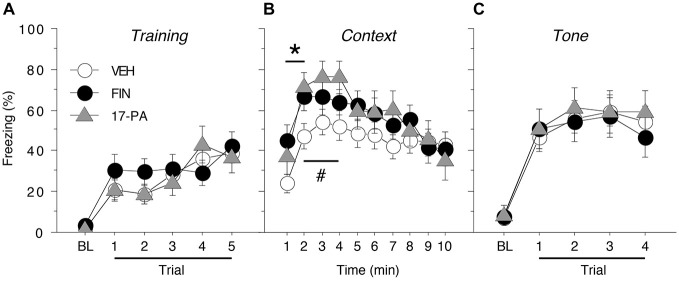
**Conditioned freezing in female rats receiving pre-test infusions of either FIN or 17-PA into the BNST. (A)** Mean percentage of freezing (± SEM) for all females during the five-trial training session (data are shown with a 3-min pre-trial period followed by five tone-shock pairings). Freezing was quantified before the first conditioning trial (baseline, BL) and during the 1-min period after each conditioning trial. **(B)** Mean percentage of freezing (± SEM) to context over 10 min 1 day after training. **(C)** Mean percentage of freezing (± SEM) to four auditory CS presentations in a novel context 2 days after training. Freezing was quantified before the first tone trial (baseline, BL) and during the 1-min period after each tone trial. *p < 0.05 FIN vs. VEH, #*p* < 0.05 17-PA vs. VEH.

Acquisition of fear conditioning did not differ between the females assigned to receive VEH, FIN, or 17-PA (Figure 3A). Levels of freezing were low prior to the start of training (<5%) and increased across the five training trials similarly for all animals. A repeated measures ANOVA with a between-subjects variable of drug group (VEH, FIN, or 17-PA) and a within-subject variable of trial (TRIAL) showed a main effect of trial (*F*_(5,250)_ = 24.7, *p* < 0.0001). There was neither a significant main effect of group (*F*_(2,50)_ = 0.31, *p* = 0.74) nor an interaction between drug group and trial (*F*_(10,250)_ = 0.98, *p* = 0.46). Thus, female rats from all groups acquired conditioned fear at similar rates and levels prior to their drug manipulations.

As shown in Figure 3B, intra-BNST infusions of either FIN or 17-PA prior to testing (2 h or 10 min, respectively) modestly, but significantly, increased levels of freezing to the conditioned context in female rats. A repeated measures ANOVA with a between-subjects variable of drug group (VEH, FIN, or 17-PA) and a within-subject variable of time (min 1–10) revealed a significant main effect of time (*F*_(9,450)_ = 14.6, *p* < 0.0001) and a significant group X time interaction (*F*_(18,450)_ = 1.81, *p* < 0.05). There was no significant main effect of drug group (*F*_(2,50)_ = 1.36, *p* = 0.27). Inspection of Figure 3B suggested that the significant group X time interaction in the ANOVA was due to increases in freezing produced by intra-BNST infusions of FIN and 17-PA in the early minutes of the context test. This impression was confirmed by planned comparisons (*p* < 0.05) of average freezing during each minute of the context text. These comparisons revealed that FIN increased freezing relative to VEH-treated rats during the first 2 min of the test; 17-PA increased freezing relative to VEH controls in minutes 2–4 of the context test. These data indicate that intra-BNST infusion of an ALLO synthesis inhibitor (FIN) or an ALLO antagonist (17-PA) enhanced the expression of contextual freezing in females.

As shown in Figure 3C, pre-test intra-BNST infusions of either FIN or 17-PA did not affect freezing during the tone test. A repeated measures ANOVA with a between-subjects variable of drug group (VEH, FIN, or 17-PA) and a within-subject variable of trial number (TRIAL) showed a main effect of trial number (*F*_(4,200)_ = 68.8, *p* < 0.0001). There was neither a significant main effect of drug group (*F*_(2,50)_ = 0.09, *p* = 0.92) nor an interaction between drug group and trial number (*F*_(8,200)_ = 0.44, *p* = 0.90). These data reveal that intra-BNST infusions of FIN or 17-PA produce a selective enhancement of contextual freezing in female rats.

## Discussion

The present results reveal that manipulations of ALLO activity within the BNST modulate the expression of conditioned contextual fear in rats. Consistent with our hypothesis, endogenous ALLO activity in males and females appears to contribute to the contextual fear phenotype in each sex. For males, in which brain ALLO levels are relatively low, augmenting BNST ALLO levels suppressed the expression of contextual freezing. In contrast, for females, in which brain ALLO levels are relatively high, reducing intra-BNST ALLO activity enhanced the expression of contextual freezing. The effects we observed were not likely due to nonspecific sensorimotor effects of the BNST drug manipulations insofar as freezing to the auditory CS was unaffected by all treatments. Together, our results suggest that ALLO in the BNST may be a critical modulator of fear behavior in both male and female rats.

By utilizing ALLO infusions into the brain, our findings extend previous work linking circulating gonadal steroid levels to contextual fear in female rats (Markus and Zecevic, 1997; Toufexis et al., 2004). In these studies, intact female rats that were conditioned and tested during proestrus, when progesterone levels are high, exhibited lower levels of contextual freezing compared to those trained and tested in other phases of the estrous cycle (Markus and Zecevic, 1997). In addition, ovariectomized female rats treated with systemic ALLO showed a significant reduction in CRH-enhanced but not fear-potentiated acoustic startle (Toufexis et al., 2004). The former is a test of “sustained” or contextual fear whereas the latter is a test of “phasic” or cued fear (Davis et al., 2010). Similar reductions have been observed in ovariectomized females treated either acutely or chronically with progesterone (Toufexis et al., 2004). By contrast, systemic treatment of ovariectomized females with medroxy-progesterone acetate, a synthetic steroid that binds to the progesterone receptor but is not metabolized to ALLO, did not affect levels of CRH-induced startle (Toufexis et al., 2004). Altogether, these data suggest that the reduced levels of context-dependent fear observed in females with elevated circulating progesterone levels may be due to the actions of its metabolite, ALLO, within the BNST.

Additional evidence supporting ALLO modulation of contextual fear can be found in studies of socially isolated mice. Male mice that have been socially isolated for three to four weeks show elevated levels of context- but not cue-specific fear (Pibiri et al., 2008). Interestingly, this change in conditioned fear behavior coincides with decreases in ALLO and 5α-reductase mRNA within components of the fear circuitry (medial prefrontal cortex, hippocampus, and basolateral amygdala). Group-housed mice showed similar increases in contextual fear following pharmacological blockade of 5α-reductase. This work lends support to the idea that reduced 5α-reductase activity in fear circuits can contribute to reduced ALLO levels in brain and concomitantly elevated contextual fear. We cannot, however, exclude the possibility that the early effects of FIN on contextual freezing in females may be due to reduced levels of the GABAergic potentiators, (3α,5α,17β)-androstane-3, 17-diol (3α-androstanediol) and (3α,5β)-3, 21-dihydroxypregnan-20-one (tetrahydrodeoxycorticosterone or THDOC), as 5α-reductase is involved in the synthesis of these compounds from 17-hydroxyandrostan-3-one (dihydrotestosterone or DHT) and 11-deoxycorticosterone (deoxycorticosterone or DOC), respectively (Reddy, 2010).

The results of the current study are consistent with the widely held view that the BNST is critical for the expression of contextual fear responses (Sullivan et al., 2004; Zimmerman and Maren, 2011) and support a role for GABAergic synaptic transmission therein. Previous work involving infusions of the GABAergic agonist, muscimol, (Fendt et al., 2003) and a GABA synthesis inhibitor (Sajdyk et al., 2008) has underscored the importance of the inhibitory neurotransmitter in regulating fear and anxiety via the BNST. Here we show for the first time that ALLO modulation of GABA_A_ receptors in the BNST may be involved in the regulation of contextual fear. While its use in behavioral paradigms is quite new, 17-PA has been well-characterized by *in vitro* and *in vivo* studies for its selective antagonism of ALLO action at GABA_A_ receptors (Mennerick et al., 2004; Kelley et al., 2007; Svensson et al., 2013). Although its antagonism of 3α-androstanediol has not been examined, 17-PA has been shown to be ineffective against 5β-reduced steroids, barbiturates, and benzodiazepines and only partially effective against THDOC (Mennerick et al., 2004; Kelley et al., 2007). Thus, the effects of the 5α-reductase inhibitor, FIN, on contextual fear in females when considered in conjunction with those of 17-PA provide support for locally available ALLO acting at GABA_A_ receptors within the BNST.

Recent human studies support links between ALLO, the BNST, and dysfunctional anxiety. Decreased levels of ALLO in CSF have been reported in premenopausal women diagnosed with PTSD compared to healthy individuals although it was not known whether this deficiency was pre-existing (Rasmusson et al., 2006). A context-specific role for the BNST in conditioned fear may apply to humans. Imaging studies have shown that nonhuman primates with trait anxious temperament have increased resting metabolism in the BNST (Oler et al., 2009) and that trait anxious human subjects exhibit exaggerated activity in the BNST during threat monitoring (Somerville et al., 2010), suggesting that “hypervigilant threat monitoring” (akin to contextual fear) may be a BNST-dependent process.

Nonetheless, the rodent data present a paradox: female rats with presumably higher levels of circulating ALLO exhibit less contextual fear and extinguish their fear responses faster than males, a pattern of results that seemingly contradicts that observed in human females (Maren et al., 1994; Gupta et al., 2001; Chang et al., 2009). Namely, although women cyclically attain higher circulating levels of ALLO than men (Genazzani et al., 1998), they exhibit a greater susceptibility to PTSD (Kessler et al., 2005). One answer to this paradox may lie in the relative propensity of males and females to extinguish fear once it is acquired. For example, studies in both rats (Gupta et al., 2001; Chang et al., 2009; Milad et al., 2009; Zeidan et al., 2011) and humans (Milad et al., 2006, 2009, 2010) reveal that low but not high estrogen levels in females retard the extinction of conditioned fear below that observed in males. Deficits in fear extinction are widely believed to contribute to the maintenance of PTSD (Pitman et al., 2012; Maren et al., 2013) and the regulation of fear extinction by gonadal steroids may be critical to increasing the vulnerability of both female rats and humans to persistent and pathological fear responding. Although it is not known how progesterone and its metabolites contribute to fear extinction, it is also possible that low levels of ALLO produce a resistance to extinction that contributes to enduring fear responses thought to underlie PTSD (Pinna, 2014). In socially isolated male mice, systemic administration of ganaxolone, a synthetic analog of ALLO, has been shown to facilitate extinction of contextual fear (Pinna and Rasmusson, 2014). Thus, cyclical variation in neuroactive steroid levels in female rats and humans might promote vulnerabilities to fear extinction, thereby contributing to disorders such as PTSD. It should be noted that ganaxolone is currently in clinical trials for treatment of PTSD^1^.

In summary, our findings suggest that ALLO within the BNST modulates contextual fear in both male and female rats. Although we cannot speak to the relative contribution of different ALLO sources (peripheral vs. brain), immunolabeling studies have shown high levels of cellular staining in the rat BNST by an anti-ALLO antibody (Saalmann et al., 2007; Cook et al., 2014), supporting the local availability of this progesterone metabolite. As previously suggested by systemic administration, ALLO promotes anxiolytic behavior in response to fear-conditioned contexts and may account for sex differences observed in contextual fear. In women, deficiencies or large variations in circulating and brain ALLO over the reproductive cycle may contribute to increased vulnerability for anxiety disorders such as PTSD. Further work on the cellular and molecular mechanisms of ALLO action in the BNST may yield insight on determinants of susceptibility to anxiety disorders both within and across genders and inform on potential avenues of pharmacological intervention.

## Author Contributions

NN and SM designed research, GMA and NN performed research, GMA and NN analyzed data, NN, GMA, and SM wrote the paper.

## Conflict of Interest Statement

The authors declare that the research was conducted in the absence of any commercial or financial relationships that could be construed as a potential conflict of interest.

## Footnotes

^1^ClinicalTrials.gov Identifier NCT01339689.
